# The burden of viral lower respiratory tract infections during the neonatal period: six-year experience at a tertiary referral hospital

**DOI:** 10.3325/cmj.2022.63.343

**Published:** 2022-08

**Authors:** Sandra Cerar, Rok Kučan, Darja Paro-Panjan, Gregor Nosan

**Affiliations:** 1 Department of Neonatology, Division of Pediatrics, University Medical Center Ljubljana, Ljubljana, Slovenia; 2 Department of Pediatric Intensive Care, Division of Pediatrics, University Medical Center Ljubljana, Ljubljana, Slovenia; 3 Department of Pediatrics, Faculty of Medicine, University of Ljubljana, Ljubljana, Slovenia

## Abstract

**Aim:**

To identify the epidemiological and clinical features of acute viral lower respiratory tract infections (LRTI) caused by respiratory syncytial virus and other respiratory viruses, and to determine the risk factors for the severe disease among neonates.

**Methods:**

We retrospectively reviewed the records of neonates aged up to 44 postconceptional weeks who were hospitalized at a tertiary referral hospital due to confirmed viral LRTI between January 2015 and December 2020.

**Results:**

Of 228 neonates with viral LRTI, one-third were born prematurely. A seasonal distribution of LRTIs from December to March was noticed, peaking in February. Forty-two percent of neonates were treated in the neonatal intensive care unit. One third of these presented with complications and needed mechanical ventilation. The most detected viruses were respiratory syncytial virus and rhinovirus. Prematurity was identified as a risk factor for worse clinical course and more complications, while rhinovirus infection was associated with an increased risk of apnea.

**Conclusions:**

The burden of respiratory syncytial virus LRTI in the neonatal period is high, although other respiratory viruses can also cause a severe respiratory disease. In preterm infants, rhinovirus infection presents an important risk factor for a severe course of LRTI with complications. Infection with two respiratory viruses leads to a more severe clinical course.

Respiratory syncytial virus (RSV) causes 12%-63% of all acute respiratory tract infections (ARTI) in children. Overall, 19%-81% of ARTI in children require hospital admission, and 2%-12% require intensive care treatment (1). The most affected population are infants younger than six months. The severity of RSV infection in this period is related to the developmental immaturity of innate and adaptive immunity manifesting as a poorly protective and dysregulated immune response, which leads to exaggerated inflammation, increased capillary permeability, bronchoconstriction, and necrosis of the respiratory epithelial cells (2,3). The concomitant insufficiency of mucociliary clearance leads to airway obstruction by thick mucus, which results in increased airway resistance, hyperinflation, and atelectasis (4).

To date, only a few studies have investigated the burden of viral LRTIs in the neonatal period. A study of children with acute bronchiolitis admitted to a pediatric intensive care unit in London over a 6-year period (2011-2016) included children up to 2 years of age (5). A Slovenian chart review also included children with bronchiolitis (2014-2015), but not exclusively newborns (6). In addition, a meta-analysis of 51 studies included newborns among older children, but focused on causative pathogens rather than the age-related characteristics (7). Therefore, the aim of this study is to identify the epidemiological and clinical features of acute viral lower respiratory tract infection (LRTI) caused by RSV and other respiratory viruses, and to determine the risk factors for a severe disease among neonates.

## PATIENTS AND METHODS

### Patients

We retrospectively reviewed the medical records of neonates aged up to 44 postconceptional weeks admitted to the University Medical Center, Ljubljana, Slovenia, due to viral LRTI between January 2015 and December 2020. The neonates were admitted to hospital based on the attending physician’s clinical decision (determined by general appearance, breathing rate, retractions, cyanosis, lethargy, room-air Sao_2_, etc). Exclusion criteria were major congenital anomalies apart from congenital heart disease and microbiologically non-confirmed viral etiology of respiratory tract infection.

The neonates were grouped according to sex, gestational age, birth weight, breastfeeding, epidemiological history, palivizumab prophylaxis, comorbidities, virus type, the month of infection, age at hospital admission, overall treatment time, and time in the neonatal intensive care unit (NICU), oxygen therapy, invasive and non-invasive ventilation, and complications (apnea, atelectasis, bacterial superinfection, sepsis).

A severe LRTI course was determined if NICU admission was needed or if disease complications (atelectasis, apnea, bacterial superinfection, sepsis) were present. The diagnosis of bacterial superinfection was based on all available clinical information (persistence of fever or a bi-phasic fever pattern, respiratory distress deterioration, higher oxygen and/or respiratory support requirements), laboratory tests (secondary elevation of inflammatory parameters, detection of bacterial causative agents), or radiographic evidence. The diagnosis of sepsis was based on the presence of bacterial superinfection and positive blood culture.

### Laboratory methods

Respiratory viruses were detected with RT-PCR in nasopharyngeal swabs or tracheal aspirates. All the samples were tested for the presence of RSV, rhinovirus (hRV), human bocavirus (hBoV), human metapneumovirus (hMPV), parainfluenza virus (PIV), influenza virus type A and B (InfV), human coronavirus (hCoV), and adenovirus (AdV). When a bacterial co-infection was suspected based on the clinical data and laboratory and radiographic findings, the patients were additionally tested for pathogenic bacteria in nasopharyngeal swab or tracheal aspirate by using aerobic culture. Aerobic blood culture was used in the case of suspected sepsis.

### Statistical analysis

Numerical data are presented as mean and standard deviation (SD), and categorical data as frequencies. The differences between independent groups in numerical variables were tested with a two-sample *t* test and a Mann-Whitney U test, while the differences in categorical variables were tested with a Fisher exact test. If the sample size was greater than 30, we used the parametric tests; if smaller than 30, we used the non-parametric tests. The *P* value <0.05 was considered statistically significant. Data were analyzed by using the R statistical software, version 4.0.3, with additional packages from the tidyverse ecosystem.

## RESULTS

During the study period, 228 neonates with acute viral LRTI were admitted to the hospital. A slight male predominance was present (Table 1). Overall, 29.5% of the neonates were born prematurely, and 83.8% were breastfed. The most frequent risk factors for contracting a respiratory virus were positive epidemiological history (87.8%) and the presence of older siblings (81.6%). A minority of the neonates (5.0%) received palivizumab, mostly due to prematurity, and the rest due to bronchopulmonary dysplasia (BPD) and congenital heart disease (6.0%). The neonates were admitted to the hospital at the mean age of 26 days; however, the disease began two to three days before that.

**Table 1:** Demographic and clinical characteristics of the study population

Characteristic	Study population (n = 228)	Preterm neonates (n = 67)	Term neonates (n = 161)	*P*
Sex, n (%)				0.770
male	128 (56.1)	39 (58.2)	89 (55.6)	
female	100 (43.9)	28 (41.8)	72 (44.4)	
Gestational age in weeks, mean (SD)	37.1 (3.7)	32.5 (3.2)	39.1 (1.1)	<0.001
Preterm, n (%)	67 (29.5)			
Birth weight in grams, mean (SD)	3052.6 (899.8)			
Breastfed, n (%)	160 (83.8)	30 (61.2)	130 (91.5)	<0.001
Palivizumab, n (%)	11 (5.0)	11 (17.7)	0 (0.0)	<0.001
Risk factors for infection, n (%)				
positive epidemiological history	172 (87.8)	44 (77.2)	128 (92.1)	0.007
siblings	168 (81.6)	49 (79.0)	119 (82.6)	0.560
Risk factors for severe disease, n (%)				
bronchopulmonary dysplasia	10 (4.4)	9 (13.4)	1 (0.6)	<0.001
congenital heart disease	9 (6.0)	5 (11.9)	4 (3.7)	0.116
Age (postmenstrual) at infection onset in days, mean (SD)	23.8 (20.3)	40.6 (28.6)	16.8 (8.8)	<0.001
Age (postmenstrual) at admission to hospital in days, mean (SD)	26.4 (20.3)	43.7 (28.7)	19.3 (8.7)	<0.001
Etiology, n (%)				
respiratory syncytial virus	181 (79.4)	39 (58.2)	142 (88.8)	<0.001
human rhinovirus	28 (12.2)	19 (28.3)	9 (5.6)	
human metapneumovirus	6 (2.6)	2 (3.0)	4 (2.5)	
parainfluenza virus	6 (2.6)	3 (4.5)	3 (1.9)	
influenza virus	5 (2.2)	3 (4.5)	2 (1.2)	
human bocavirus	4 (1.8)	1 (1.5)	3 (1.9)	
human coronavirus	3 (1.3)	0 (0)	3 (1.9)	
two respiratory viruses	5 (2.2)	2 (3.0)	3 (1.9)	
Complications, n (%)				
apnea	93 (40.8)	39 (58.2)	54 (33.8)	0.001
atelectasis	74 (32.6)	27 (40.3)	46 (28.9)	0.119
bacterial superinfection	102 (44.9)	39 (58.2)	62 (39.0)	0.009
sepsis	14 (6.2)	6 (9.0)	8 (5.0)	0.363
Length of hospitalization in days, mean (SD)	9.2 (7.0)	12.7 (10.4)	7.7 (4.2)	<0.001
Length of NICU hospitalization in days, mean (SD)	3.3 (4.8)	5.5 (6.0)	2.3 (3.8)	<0.001
Treatment, n (%)				
oxygen	199 (87.3)	59 (88.1)	139 (86.9)	1.000
non-invasive ventilation	100 (43.9)	36 (53.7)	63 (39.4)	0.057
invasive ventilation	74 (32.5)	34 (50.7)	39 (24.4)	<0.001
Length of treatment in days, mean (SD)				
oxygen	4.5 (5.2)	5.8 (7.9)	3.9 (3.4)	0.013
non-invasive ventilation	1.4 (2.2)	1.9 (2.8)	1.1 (1.9)	0.023
invasive ventilation	2.4 (4.0)	4.1 (5.3)	1.6 (3.0)	<0.001

A typical seasonal distribution of LRTIs was observed (Figure 1). Most neonates were admitted from December to March, with a peak in February. The median hospital stay duration was nine days. More than 40% of neonates were treated in the NICU, and one third of this needed mechanical ventilation. A similar proportion of neonates had complications, including apnea, atelectasis, and bacterial superinfection.

In 79.4% of participants, the causative viral pathogen was RSV, followed by hRV in 12.2%, hMPV and PIV in 2.6% each, InfV in 2.2%, hBoV in 1.8%, and hCoV in 1.3% of participants. Two respiratory viruses were detected in 2.2% of neonates, most commonly RSV and hRV.

In the pandemic year 2020, the overall number of newborns with LRTI and newborns contracting RSV LRTI declined significantly. Soon after the lockdown, no more RSV cases were detected, and by the end of the year some sporadic non-RSV cases had occurred (hRV, hMPV). The decline in LRTI due to RSV in 2020 appeared earlier, and the winter peak was absent.

In cases of bacterial superinfection, pneumonia, or sepsis, the detected bacterial causative agents were *Streptococcus pneumoniae, Haemophilus influenzae, Staphylococcus epidermidis, Mycoplasma pneumoniae, Moraxella catarrhalis, Escherichia coli, and Klebsiella pneumoniae.*

### Prematurity

Preterm neonates with viral LRTI were older at the onset of the infection and had a worse clinical course of the disease, needed longer hospital stay, more frequent NICU treatment, and non-invasive and invasive ventilation, which was needed for a longer time (Table 1). Moreover, they had a higher incidence of apnea and bacterial superinfection compared with term neonates. Interestingly, preterm neonates were less likely than term neonates to contract a RSV infection.

### Respiratory syncytial virus and rhinovirus infection

Neonates with RSV LRTI were younger at the onset of the infection and at hospital admission, were more commonly breastfed, and had a higher incidence of atelectasis than neonates with a non-RSV LRTI. Four preterm neonates were admitted to the hospital due to RSV LRTI, despite receiving palivizumab prophylaxis. As mentioned, preterm neonates more commonly had non-RSV infections, especially hRV infection. Another difference was a higher incidence of apnea in the hRV group compared with the non-hRV group (Table 2).

**Table 2:** Comparison of clinical characteristics, treatment, and complications of neonates with respiratory syncytial virus (RSV), non-RSV, human rhinovirus (hRV), and non-hRV lower respiratory tract infection

Characteristic	RSV infection (n = 181)	Non-RSV infection (n = 47)	*P* value	hRV infection (n = 28)	Non-hRV infection (n = 200)	*P*
Sex, n (%)			0.624			0.419
male	100 (55.2)	28 (95.6)		18 (64.3)	110 (55.0)	
female	81 (44.8)	19 (40.4)		10 (35.7)	90 (45.0)	
Gestational age <37 weeks, n (%)	39 (21.5)	28 (60.9)	<0.001	19 (67.9)	48 (24.1)	<0.001
Breastfed, n (%)	141 (89.8)	19 (55.9)	<0.001	13 (65.0)	147 (86.0)	0.025
Palivizumab, n (%)	4 (2.3)	7 (15.6)	0.002	6 (21.4)	5 (2.6)	<0.001
Risk factors for infection, n (%)						
positive epidemiological history	143 (90.5)	29 (76.3)	0.026	20 (83.3)	152 (88.4)	0.505
siblings	132 (80.0)	36 (87.8)	0.368	23 (88.5)	145 (80.6)	0.426
Risk factors for severe disease						
bronchopulmonary dysplasia	5 (2.8)	5 (10.6)	0.034	3 (10.7)	7 (3.5)	0.110
congenital heart disease	6 (4.8)	3 (11.5)	0.186	3 (15.0)	6 (4.6)	0.099
Age (postmenstrual) at infection onset in days, mean (SD)	21.1 (15.8)	34.5 (30.1)	<0.001	37.5 (34.0)	21.9 (16.8)	0.066
Age (postmenstrual) at hospitalization in days, mean (SD)	23.6 (15.7)	37.7 (30.4)	<0.001	40.3 (34.8)	24.5 (16.6)	0.059
Length of hospitalization in days, mean (SD)	8.9 (5.4)	10.5 (11.2)	0.144	10.5 (9.8)	9.0 (6.6)	0.830
Length of NICU hospitalization in days, mean (SD)	3.1 (4.7)	3.9 (5.1)	0.343	3.9 (5.6)	3.2 (4.7)	0.874
Treatment						
oxygen	159 (87.8)	40 (85.1)	0.626	24 (85.7)	175 (87.5)	0.764
non-invasive ventilation	80 (44.2)	20 (42.6)	0.870	10 (35.7)	64 (45.0)	0.419
invasive ventilation	56 (30.9)	18 (38.3)	0.383	10 (35.7)	64 (32.0)	0.673
Length of treatment in days, mean (SD)						
oxygen	4.4 (4.7)	4.8 (6.8)	0.658	4.3 (4.4)	4.5 (5.3)	0.624
non-invasive ventilation	1.4 (2.1)	1.2 (2.4)	0.679	1.0 (2.0)	1.4 (2.2)	0.327
invasive ventilation	2.2 (4.0)	2.9 (4.2)	0.306	3.2 (4.7)	2.2 (3.9)	0.398
Complications						
apnea	67 (37.0)	26 (55.3)	0.030	17 (60.7)	76 (38.0)	0.025
atelectasis	62 (34.4)	12 (25.5)	0.296	9 (32.1)	65 (32.7)	1.000
bacterial superinfection	81 (45.0)	21 (44.7)	1.000	13 (46.4)	89 (44.7)	1.000
sepsis	10 (5.6)	4 (8.5)	0.496	3 (10.7)	11 (5.5)	0.390

### Infection with two respiratory viruses

Two respiratory viruses were detected in 5 (2.2%) neonates with LRTI. RSV was one of the causative pathogens in all except one neonate (92.9%), followed by hRV. Neonates with LRTI caused by two viruses required longer hospital stay (11.1 vs 9.7 days, *P* = 0.021), longer NICU stay (5.6 vs 3.1 days, *P* = 0.044), and more frequent invasive ventilation (57.1 vs 30.8%, *P* = 0.072) with a longer course (4.3 vs 2.2 days, *P* = 0.033).

### Palivizumab

All 11 palivizumab recipients were born prematurely and had a higher incidence of BPD (45.5 vs 1.9%, *P* < 0.001). They were older at the onset of the infection (63.7 vs 21.4 days, *P* < 0.001), had a higher incidence of sepsis (27.3 vs 5.3%, *P* = 0.025) and apnea (72.7 vs 38.6%, *P* = 0.030), more frequently required invasive ventilation (63.6 vs 29.0%, *P* = 0.038), and needed a longer course of non-invasive (3.7 vs 1.2 days, *P* = 0.013) and invasive ventilation (5.1 vs 20 days, *P* = 0.006) than palivizumab non-recipients.

### Breastfeeding

Almost 84% of neonates were breastfed, and a large majority of these were term (81.2 vs 18.8%, *P* < 0.001). Breastfed neonates were also younger at the infection onset (22.4 vs 32.3 days, *P* = 0.014), needed shorter hospital stay (8.1 vs 12.2 days, *P* = 0.002), and less frequently needed invasive ventilation (25.6 vs 74.4%, *P* = 0.049) than non-breastfed neonates.

### Month of contracting the infection

The analysis of LRTI seasonality revealed some intriguing results, especially for the winter season. Neonates who contracted LRTI in January were more frequently premature (42.9 vs 24.4%, *P* = 0.009), had BPD (9.5 vs 2.4%, *P* = 0.029), received palivizumab (14.8 vs 1.2%, *P* < 0.001), needed a longer NICU stay (4.4 vs 2.9 days, *P* = 0.012) and hospital stay (10.8vs. 8.6 days, *P* = 0.026), and had a higher incidence of complications, such as atelectasis (46.0 vs 27.4%, *P* = 0.011) and apnea (52.4 vs 36.4%, *P* = 0.035), compared with neonates who contracted the infection in other months.

The overall incidence of LRTI was highest in February, especially due to RSV (83.9 vs 74.5%, *P* = 0.009). Neonates who contracted LRTI in February were younger at the onset of the disease (17.5 vs 26.9 days, *P* < 0.001), but the clinical course of LRTI during this month in certain aspects was milder compared with other months.

## DISCUSSION

Though this study confirmed a high burden of RSV, which is known to be prevalent in children under two years of age, the main finding was that other respiratory viruses also caused severe respiratory disease. Additionally, the study found that in preterm infants a non-RSV infection may be a serious risk factor for a severe course of LRTI. To the best of our knowledge, this is one of the few studies to focus on this vulnerable period of life, in which prenatal and perinatal risk factors and an immature immunological system interact with the pathogen properties and clinical course of the disease.

The high burden of RSV infection is exemplified by more than 3/4 of LRTI cases caused by the virus in this study. All other respiratory viruses together were responsible for less than 1/4 of the LRTI cases, predominantly hRV infection, accounting for 12.2% of cases, followed by hMPV, PIV, InfV, hBoV, and hCoV. Other comparable studies, although conducted in older children, showed a similar distribution of causative viral agents. In these studies, RSV accounted for 60%-88% of viral LRTI in children below 12 months of age, followed by hRV, InfV, HCoV, and hMPV (5,6,8). Similarly, in a meta-analysis of 51 studies, RSV was the most detected virus in children under two years of age (59%), followed by hRV (19%), hBoV, AdV, hMPV, PIV, InfV, and hCoV (7). RSV was also the predominant virus detected in multiple-etiology cases, and the most common couple was RSV and hRV. The latter finding was also consistent with this study.

RSV LRTI in children is an important reason for hospital admission. A study from England showed that most hospital admissions due to RSV infection occurred in the first three months of life (9). A recent meta-analysis of acute RSV LRTI in children younger than five years, encompassing 58 countries, revealed that 45% of infants with RSV LRTI who were younger than one year needed hospital admission (10). At the same time, only 23% of infants younger than six months with InfV infection needed hospital admission (11).

In children under two years, RSV causes more severe LRTI than other respiratory viruses, and premature neonates are the most vulnerable subgroup of these children (8,9). A combination of weaker respiratory epithelium protection and immune response could permit greater viral replication, and consequently increased disease severity in preterm neonates (2,4). However, most infants hospitalized due to RSV LRTI are born at term and have no risk factors for the severe course of the disease (9).

Preterm neonates had a worse clinical course of LRTI than term neonates, as they needed longer hospital stay, more frequent NICU treatment, and non-invasive ventilation, and finally more frequent and longer invasive ventilation. They were also more likely to have apnea and secondary bacterial superinfection. A study investigating LRTI infections with hMPV, RSV, and PIV in term and preterm infants similarly revealed a more severe disease course in the preterm group (12).

However, our study showed preterm neonates having a lower incidence of RSV infection and a higher incidence of non-RSV, particularly hRV, infection compared with term neonates. Non-RSV infection seems to be a significant risk factor for a severe course of LRTI in this subgroup. Furthermore, 58% of preterm neonates with hRV LRTI had apneas, 46% had bacterial superinfection, and one neonate died from sepsis. These findings are consistent with other studies showing hRV as an important pathogen and risk factor for a severe respiratory disease in preterm neonates. They also confirm that young age and prematurity are the main risk factors for apnea, regardless of the causative respiratory virus (13,14). Additionally, apneas could be related to the pathophysiology of the LRTI and not to the pathogen itself (15). On the other hand, extreme prematurity could lead to increased airway secretion of Th2 and Th17 cytokines during hRV infections, which closely resembles the inflammation in asthma, and could be associated with increased respiratory morbidity in the first two years of life (16,17).

Except RSV and hRV, the study did not find that any other respiratory virus significantly affected the severity of LRTI in neonates, probably simply due to the low incidence of these viruses. Although *Bordetella pertussis* (BP) infection is common in young infants hospitalized for acute bronchiolitis, mostly as a coinfection with respiratory viruses, and although it is difficult to differentiate viral bronchiolitis from pertussis based on clinical findings and unspecific laboratory parameters, BP infection was not routinely ruled out in our study. In fact, a single case of BP monoinfection was found, but due to the absence of viral coinfection the case was excluded from the analysis. A targeted testing for BP would probably demonstrate a higher presence of this bacterium. However, BP coinfection usually does not affect the clinical severity in infants hospitalized due to acute viral bronchiolitis (18,19).

Preterm neonates were more than twice as old at the onset of LRTI as term neonates. This could be explained by their longer primary hospital stay due to prematurity and their consequent delayed environmental exposure to respiratory viruses. Although the immune response of preterm and term neonates differs significantly at birth, the main differences diminish at three months (20). Therefore, the age difference between preterm and term neonates was still too small from the immunological point of view to claim that preterm neonates were immunologically similar to their term peers.

Remarkably, the study did not confirm the vastly different clinical course of LRTI caused by RSV and other respiratory viruses observed in other studies. Very few available studies investigate the differences in the clinical course of RSV and non-RSV LRTI in the neonatal period. One of them similarly reported no differences between the groups in the occurrence of cough, fever, wheezing, dyspnea, cyanosis, and refusal to feed, although neonates with RSV presented more frequently with tachypnea, moist rales, and extended hospital stay (21). However, studies comparing the clinical course of LRTI in older children (below two years of age) showed that the length of hospital stay, intensive care unit hospitalization, and mechanical ventilation was longer in children with RSV infection (22).

In our study, the number of palivizumab recipients was rather low (5.0%), and most of them (63.6%) contracted non-RSV LRTI, which in a way could be interpreted as effective RSV prophylaxis. However, four neonates receiving palivizumab (exclusively due to prematurity) did contract RSV infection. All four cases occurred during December and February, when the environmental viral load reaches its peak and they all had siblings. All except one were breastfed. The first two patients were extremely preterm twins (27 weeks’ gestation) who received three doses of palivizumab. The first twin needed three days of non-invasive ventilation; the second one had bacterial pneumonia with atelectasis and required nine days of invasive ventilation and additional nine days of non-invasive ventilation. The last two neonates were born after 31 weeks’ gestation and both received one dose of palivizumab. The third neonate required eight days of invasive ventilation, also due to bacterial pneumonia. The fourth neonate had the mildest clinical course as he required only four-day additional oxygen treatment. Palivizumab prophylaxis is mainly limited to selected high-risk infants for the first RSV season (23). It generally prevents RSV infection and the severe course of LRTI, unfortunately not entirely, as was also observed in this study. Therefore, all other epidemical preventive measures should still be followed, at least until a more efficient monoclonal antibody or vaccine arrives (24).

Breastfeeding, probably due to a wide range of immunomodulatory constituents of human milk, provides a broad anti-inflammatory defense for the infant against diarrhea, respiratory infections, necrotizing enterocolitis, otitis media, and possibly also against urinary tract infections, allergies, and other immunological diseases (25-28). This study also confirms the beneficial effect of human milk on the clinical course of LRTI in neonates as breastfeeding evidently shortened the hospital stay and reduced the need for invasive ventilation. Obviously, most breastfed neonates were born at term, so prematurity could also have significantly influenced the clinical course. Still, the question remains why respiratory viruses affect neonates to such an extent, despite the high proportion of breastfed infants. One study suggests that mothers of infants with LRTI produce lower IgG concentrations in their milk, which could play an important role in disease severity (29).

Acute viral LRTI is a seasonal disease, appearing most frequently in epidemics during the winter months in the northern hemisphere (1,7). This is consistent with the findings of this study, which showed a typical seasonal pattern of LRTI in neonates caused by all respiratory viruses, especially RSV. According to the data from Slovenian National Institute of Public Health, the RSV epidemics in 2015 to 2019 started in December, peaked in February, and ended in April (30). The occurrence of neonatal RSV LRTI clearly followed this national pattern.

The monthly variability of the characteristics and clinical course of neonates with LRTI was evident during winter months. Neonates hospitalized in January were more likely to be preterm, needed longer hospital stay, and had a more severe disease course and a higher incidence of complications. Most neonates hospitalized in February were born at term and were younger at the onset of the infection. There was a remarkably high incidence of RSV LRTI, apparently due to an increased environmental viral load, which possibly also caused infection of chronologically younger individuals. Interestingly, in March, the third most affected month of the year, no significant differences were found.

Notably, this study was conceived before the COVID-19 era but includes the data for the entire 2020, in the beginning of which the pandemic started. Although no SARS-CoV-2-positive newborn was detected by the end of the study, the epidemiology of viral respiratory infections during the pandemic year was significantly influenced by preventive epidemiological measures. Low level of respiratory infections, atypical seasonality with earlier decline of RSV LRTIs, and the absence of winter peak was observed. In contrast to RSV, which was not detected after the lockdown, despite their overall decline, some individual non-RSV cases were identified throughout 2020.

The main limitations of our study were the retrospective methodology and monocentric data sampling. However, the study improves the insight into neonatal viral LRTI and offers future research suggestions for temporal and geographical sample expansion and comparison of the pre-COVID-19 and post-COVID-19 pandemic era.

In conclusion, the burden of RSV LRTI in the neonatal period is high, although infection with other respiratory viruses can also cause severe respiratory disease. Especially in preterm infants hRV presents an important risk factor for a severe course of LRTI with complications. Infection with two respiratory viruses leads to a more severe clinical course. February is the most affected month, with the highest incidence of RSV infections. Palivizumab effectively prevents RSV infection and the severe course of LRTI, although not entirely.

**Table:** Figure 1. Monthly occurrence of viral lower respiratory tract infections due to respiratory syncytial virus (RSV) and non-RSV etiology

## References

[1] Van de Steen O Miri F Gunjaca M Klepac V Gross B Notario G The burden of severe respiratory syncytial virus disease among children younger than 1 year in Central and Eastern Europe. Infect Dis Ther 2016 5 125 37 10.1007/s40121-016-0109-y 27174177 PMC4929088

[2] Lambert L Culley FJ Innate immunity to respiratory infection in early life. Front Immunol 2017 8 1570 10.3389/fimmu.2017.01570 29184555 PMC5694434

[3] Rossi GA Colin AA Infantile respiratory syncytial virus and human rhinovirus infections: Respective role in inception and persistence of wheezing. Eur Respir J 2015 45 774 89 10.1183/09031936.00062714 25359340

[4] Derscheid RJ Ackermann MR The innate immune system of the perinatal lung and responses to respiratory syncytial virus infection. Vet Pathol 2013 50 827 41 10.1177/0300985813480216 23528938

[5] Ghazaly M Nadel S Characteristics of children admitted to intensive care with acute bronchiolitis. Eur J Pediatr 2018 177 913 20 10.1007/s00431-018-3138-6 29654399 PMC5958152

[6] Praznik A Vinšek N Prodan A Erčulj V Pokorn M Mrvič T Risk factors for bronchiolitis severity: A retrospective review of patients admitted to the university hospital from central region of Slovenia. Influenza Other Respir Viruses 2018 12 765 71 10.1111/irv.12587 29944781 PMC6185887

[7] Kenmoe S Kengne-Nde C Ebogo-Belobo JT Mbaga DS Modiyinji AF Njouom R Systematic review and meta-analysis of the prevalence of common respiratory viruses in children < 2 years with bronchiolitis in the preCOVID-19 pandemic era. PLoS One 2020 15 e0242302 10.1371/journal.pone.0242302 33180855 PMC7660462

[8] Pichler K Assadian O Berger A Viral respiratory infections in the neonatal intensive care unit-a review. Front Microbiol 2018 9 2484 10.3389/fmicb.2018.02484 30405557 PMC6202802

[9] Murray J Bottle A Sharland M Modi N Aylin P Majeed A Risk factors for hospital admission with RSV bronchiolitis in England: A population-based birth cohort study. PLoS One 2014 9 e89186 10.1371/journal.pone.0089186 24586581 PMC3935842

[10] Li Y Johnson EK Shi T Campbell H Chaves SS Commaille-Chapus C National burden estimates of hospitalisations for acute lower respiratory infections due to respiratory syncytial virus in young children in 2019 among 58 countries: a modelling study. Lancet Respir Med 2021 9 175 85 10.1016/S2213-2600(20)30322-2 32971018

[11] Wang X Li Y O’Brien KL Madhi SA Widdowson MA Byass P Global burden of respiratory infections associated with seasonal influenza in children under 5 years in 2018: a systematic review and modelling study. Lancet Glob Health 2020 8 e497 510 10.1016/S2214-109X(19)30545-5 32087815 PMC7083228

[12] El Basha NR Marzouk H Sherif MM El Kholy AA Prematurity, a significant predictor for worse outcome in viral bronchiolitis: a comparative study in infancy. J Egypt Public Health Assoc 2019 94 15 10.1186/s42506-019-0015-8 32218612 PMC7091660

[13] Kathryn Miller E Bugna J Libster R Shepherd BE Scalzo PM Acosta PL Human rhinoviruses in severe respiratory disease in very low birth weight infants. Pediatrics 2012 129 e60 7 10.1542/peds.2011-0583 22201153 PMC3255465

[14] Ricart S Rovira N Garcia-Garcia JJ Pumarola T Pons M Muñoz-Almagro C Frequency of apnea and respiratory viruses in infants with bronchiolitis. Pediatr Infect Dis J 2014 33 988 90 10.1097/INF.0000000000000365 24797994

[15] Wishaupt J Van Den Berg E Van Wijk T Van Der Ploeg T Versteegh F Hartwig N Paediatric apnoeas are not related to a specific respiratory virus, and parental reports predict hospitalisation. Acta Paediatr 2016 105 542 8 10.1111/apa.13375 26910649 PMC7159689

[16] Perez GF Kurdi B Megalaa R Pancham K Huseni S Isaza N Phenotypical characterization of human rhinovirus infections in severely premature children. Pediatr Neonatol 2018 59 244 50 10.1016/j.pedneo.2017.04.008 29033350 PMC5871590

[17] Perez GF Pancham K Huseni S Jain A Rodriguez-Martinez CE Preciado D Rhinovirus-induced airway cytokines and respiratory morbidity in severely premature children. Pediatr Allergy Immunol 2015 26 145 52 10.1111/pai.12346 25640734 PMC5542573

[18] Gökçe Ş Kurugöl Z Şöhret Aydemir S Çiçek C Aslan A Koturoğlu G Bordetella Pertussis infection in hospitalized infants with acute bronchiolitis. Indian J Pediatr 2018 85 189 93 10.1007/s12098-017-2480-4 29076101 PMC7090534

[19] Frassanito A Nenna R Nicolai A Pierangeli A Tozzi AE Stefanelli P Infants hospitalized for Bordetella pertussis infection commonly have respiratory viral coinfections. BMC Infect Dis 2017 17 492 10.1186/s12879-017-2567-6 28701160 PMC5506634

[20] Olin A Henckel E Chen Y Lakshmikanth T Pou C Mikes J Stereotypic immune system development in newborn children. Cell 2018 174 1277 92 10.1016/j.cell.2018.06.045 30142345 PMC6108833

[21] Lu L Yan Y Yang B Xiao Z Feng X Wang Y Epidemiological and clinical profiles of respiratory syncytial virus infection in hospitalized neonates in Suzhou, China. BMC Infect Dis 2015 15 431 10.1186/s12879-015-1155-x 26470889 PMC4608146

[22] García CG Bhore R Soriano-Fallas A Trost M Chason R Ramilo O Risk factors in children hospitalized with RSV bronchiolitis versus non-RSV bronchiolitis. Pediatrics 2010 126 10.1542/peds.2010-0507 21098154 PMC3761792

[23] Resch B Product review on the monoclonal antibody palivizumab for prevention of respiratory syncytial virus infection. Hum Vaccin Immunother 2017 13 1 12 10.1080/21645515.2017.1337614 28605249 PMC5612471

[24] Sala KA Moore A Desai S Welch K Bhandari S Carroll CL Factors associated with disease severity in children with bronchiolitis. J Asthma 2015 52 268 72 10.3109/02770903.2014.956893 25158108

[25] Dixon D-L The role of human milk immunomodulators in protecting against viral bronchiolitis and development of chronic wheezing illness. Children (Basel) 2015 2 289 304 10.3390/children2030289 27417364 PMC4928768

[26] Bryan DL Hart PH Forsyth KD Gibson RA Immunomodulatory constituents of human milk change in response to infant bronchiolitis. Pediatr Allergy Immunol 2007 18 495 502 10.1111/j.1399-3038.2007.00565.x 17680907

[27] Ogawa J Sasahara A Yoshida T Sira MM Futatani T Kanegane H Role of transforming growth factor-β in breast milk for initiation of IgA production in newborn infants. Early Hum Dev 2004 77 67 75 10.1016/j.earlhumdev.2004.01.005 15113633

[28] Hanson LÅ. Session 1: Feeding and infant development breast-feeding and immune function - Symposium on “Nutrition in early life: New horizons in a new century.” In: Proceedings of the Nutrition Society. Vol 66. Proc Nutr Soc. 2007:384-96. 10.1017/S0029665107005654 17637091

[29] Li C Liu Y Jiang Y Xu N Lei J Immunomodulatory constituents of human breast milk and immunity from bronchiolitis. Ital J Pediatr 2017 43 1 7 10.1186/s13052-017-0326-3 28257632 PMC5347836

[30] Sočan M, Steiner Rihtar S, Prosenc K, Grilc E. Surveillance of respiratory syncytial virus in 2019/2020 season in Slovenia. E-newsletter on Communicable Diseases and Environmental Health No. 6.

